# P-2001. Integrating a host biomarker and a large language model for diagnosis of lower respiratory tract infection

**DOI:** 10.1093/ofid/ofaf695.2165

**Published:** 2026-01-11

**Authors:** Natasha Spottiswoode, Hoang Van Phan, Emily Lydon, Victoria Chu, Adolfo Cuesta, Alexander Kazberouk, Natalie Richmond, Carolyn Calfee, Chaz Langelier

**Affiliations:** University of California, San Francisco, San Francisco, CA; University of California San Francisco, San Francisco, California; University of California San Francisco, San Francisco, California; University of California, San Francisco, San Francisco, CA; University of California San Francisco, San Francisco, California; University of California San Francisco, San Francisco, California; University of California San Francisco, San Francisco, California; University of California San Francisco, San Francisco, California; University of California San Francisco, San Francisco, California

## Abstract

**Background:**

Lower respiratory tract infections (LRTIs) are a leading cause of mortality worldwide and can be difficult to diagnose in critically ill patients, as non-infectious causes of respiratory failure can present with similar clinical features.

**Figure 1.**
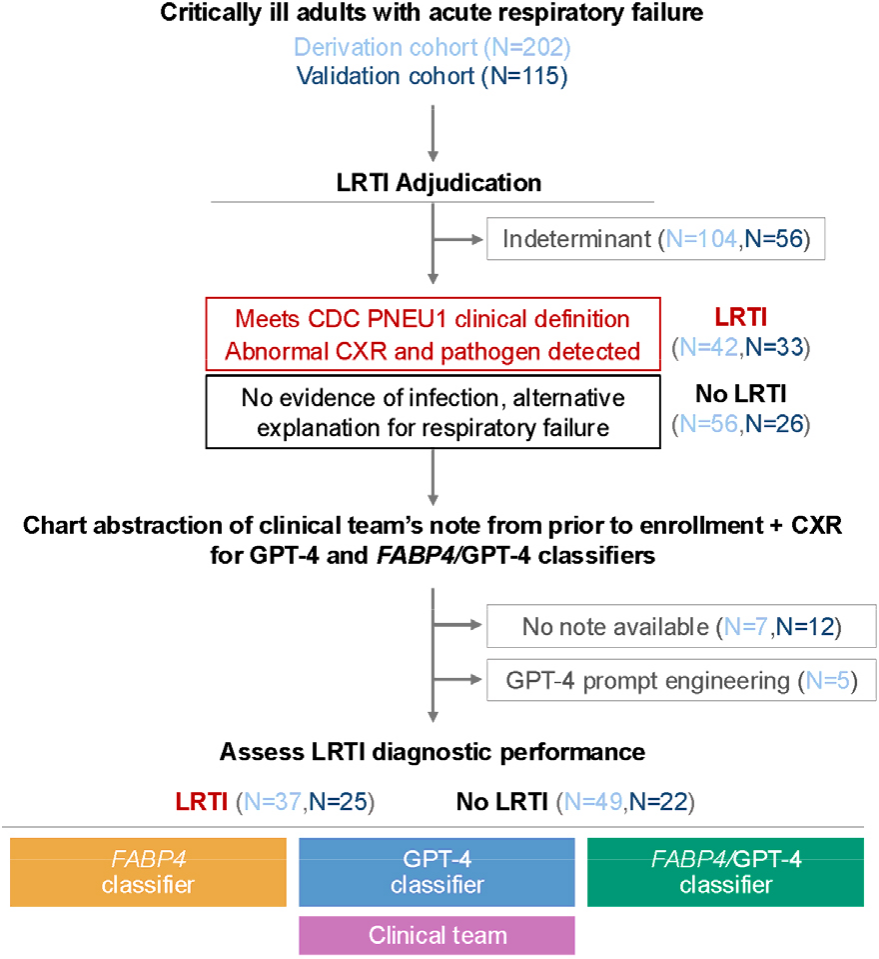
Study flow diagram and overview. Abbreviations: LRTI = lower respiratory tract infection; RNA-seq = RNA sequencing; CXR = chest X ray, FABP4 = gene encoding fatty acid binding protein 4; CDC = U.S. Centers for Disease Control and Prevention; GPT-4 = Generative Pre-trained Transformer 4.

**Figure 2.**
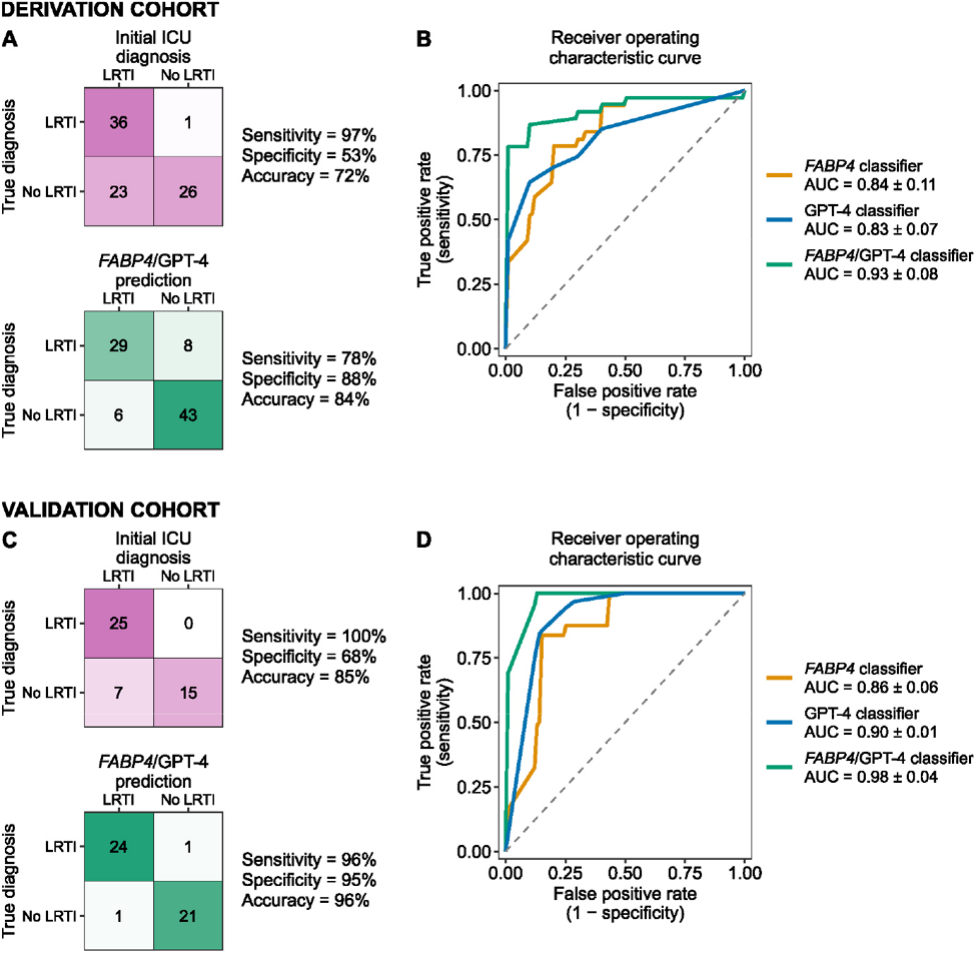
Performance of FABP4, GPT-4 and integrated LRTI diagnostic classifiers in the derivation and validation cohorts. A) Confusion matrices for initial ICU diagnosis and the integrated FABP4/GPT-4 classifier in the derivation cohort. B) Receiver operating characteristic curves from GPT-4 classifier, FABP4 classifier, and integrated FABP4/GPT-4 classifier in the derivation cohort. C) Confusion matrices for initial ICU diagnosis and the integrated FABP4/GPT-4 classifier in the validation cohort. D) Receiver operating characteristic curves from GPT-4 classifier, FABP4 classifier, and integrated FABP4/GPT-4 classifier in the validation cohort. In panels A and C, the classifiers output an LRTI diagnosis if the patients had a predicted out-of-fold LRTI probability of 50% or higher. In panels B and D, the area under the curves (AUCs) are presented as mean +/- standard deviation.

**Methods:**

We developed a LRTI diagnostic method combining the pulmonary transcriptomic biomarker *FABP4* with electronic medical record (EMR) text assessment using the large language model Generative Pre-trained Transformer 4 (GPT-4). We evaluated this approach in a prospective cohort of critically ill adults with acute respiratory failure from whom tracheal aspirate *FABP4* expression was measured by RNA sequencing. Patients with LRTI or non-infectious conditions were identified using retrospective, multi-physician clinical adjudication. We then confirmed our findings by applying this method to an independent validation cohort of 115 adults with acute respiratory failure (Figure 1). Additionally, we compared GPT-4 diagnostic performance to physicians given the identical EMR information.

**Figure 3.**
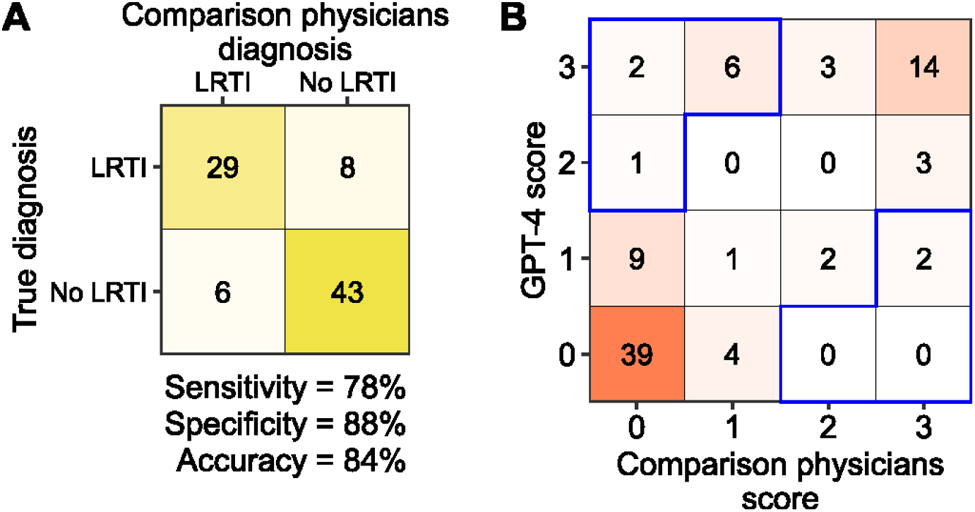
Comparison of GPT-4 performance to physicians provided the same EMR data from the LRTI derivation cohort. A) Confusion matrix of diagnosis by three GPT-4 comparison physicians who received the same prompt and data as GPT-4. B) Comparison of GPT-4 LRTI scores as compared to physicians. In Panel B, X-axis depicts the number of times GPT-4 diagnosed LRTI out of 3, Y-axis shows the number of times the physicians called LRTI out of 3. Blue boxes indicate instances in which GPT-4 diagnoses were most discordant with comparison physicians (the scores differ by 2 or more).

**Results:**

In the derivation cohort, a combined classifier incorporating *FABP4* expression and GPT-4–assisted EMR analysis achieved an AUC of 0.93 (±0.08) and an accuracy of 84%, outperforming *FABP4* expression alone (AUC 0.84 ± 0.11) and GPT-4–based analysis alone (AUC 0.83 ± 0.07; Figure 2). By comparison, the primary medical team’s admission diagnosis had an accuracy of 72%. In the validation cohort, the combined classifier yielded an AUC of 0.98 (±0.04) and an accuracy of 96%. In comparison to human physicians, GPT-4 over-indexed on chest X-ray reads and under-emphasized notes from the clinical team (Figure 3).

**Conclusion:**

Integrating a host transcriptional biomarker with EMR text analysis using a large language model may offer a promising new approach to improving the diagnosis of LRTIs in critically ill adults. As a next step, we plan to extend this approach to other critical illness infectious syndromes, such as sepsis.

**Disclosures:**

All Authors: No reported disclosures

